# Supercharging the metabolic engine: mitochondrial engineering strategies for CAR-T persistence in solid tumors

**DOI:** 10.3389/fimmu.2026.1822668

**Published:** 2026-05-08

**Authors:** Soohyun Chun, Sanghyeon Yu, Hyun Gu Lee, Man S. Kim

**Affiliations:** 1 Translational-Transdisciplinary Research Center, Medical Science Research Institute, Kyung Hee University Hospital at Gangdong, College of Medicine, Kyung Hee University, Seoul, Republic of Korea; 2 Department of Medicine, Kyung Hee University College of Medicine, Seoul, Republic of Korea; 3 Department of Biomedical Science and Technology, Graduate School, Kyung Hee University, Seoul, Republic of Korea; 4 Center for Space Biomedical Science, NEXUS Institute, Kyung Hee University, Yongin, Republic of Korea; 5 Department of Surgery, Kyung Hee University Hospital at Gangdong, Kyung Hee University College of Medicine, Seoul, Republic of Korea

**Keywords:** CAR-T cell therapy, immunometabolism, metabolic reprogramming, metabolic-epigenetic axis, mitochondrial engineering, solid tumors, T cell exhaustion, tumor microenvironment

## Abstract

Chimeric antigen receptor (CAR)-T cell therapy has achieved remarkable success in hematological malignancies, yet its efficacy in solid tumors is severely limited by the metabolically hostile tumor microenvironment (TME). Within this landscape, CAR-T cells undergo rapid functional exhaustion driven by mitochondrial dysfunction and metabolic insufficiency. This mini-review synthesizes emerging mitochondrial engineering strategies designed to restore metabolic fitness and persistence. We first examine the newly identified metabolic-epigenetic axis, where the pathological mitochondrial translocation of *P4HA1* and the concomitant accumulation of oncometabolite succinate lock T cells in an exhausted state, and discuss how targeting this pathway restores progenitor subsets. Furthermore, we explore genetic reprogramming approaches, including “Envirotune” platforms that couple hypoxia-sensing elements (HRE) with enhanced glutamine transport (*SLC38A2*), and CRISPR-identified targets such as *RHOG* and *FAS* that prevent fratricide and apoptosis to preserve effector pools. Finally, we highlight the frontier of organelle medicine, focusing on intercellular mitochondrial transfer via tunneling nanotubes (TNTs) mediated by Talin-2, and emerging computational strategies to detect mitochondrial hijacking risk. By integrating these metabolic interventions, next-generation CAR-T cells can be engineered to overcome the TME’s metabolic barriers, transforming them from transient effectors into long-lived, highly effective therapeutic agents.

## 1 Introduction

Chimeric antigen receptor (CAR)-T cell therapy has redefined the treatment of B-cell malignancies. However, its success in solid tumors is limited by the hostile tumor microenvironment (TME), which induces profound dysfunction known as exhaustion (1–3). While checkpoint blockade (e.g., anti-PD-1) releases molecular brakes, it is often insufficient in the metabolically desolate landscape of solid tumors (4). Consequently, the field has shifted toward immunometabolism, identifying mitochondrial fitness not merely as a fuel source but as a central checkpoint governing T cell fate and antitumor potency (5, 6).

### 1.1 The tripartite metabolic liability of the TME

Upon infiltrating a solid tumor, CAR-T cells encounter a “metabolic battlefield” defined by hypoxia, nutrient deprivation, and oncometabolite accumulation (4, 7). This environment does not simply starve T cells but actively drives maladaptive reprogramming into a fragmented mitochondrial state associated with terminal exhaustion (6, 8, 9).

### 1.2 Mitochondria: from powerhouse to signaling hub

Mitochondria are increasingly recognized as the orchestrators of T cell differentiation. Their morphology—governed by the balance between fusion (mediated by *Opa1*) and fission (mediated by *Drp1*)—acts as an intrinsic determinant of longevity (10). “Stem-like” central memory T cells (T_CM_), correlated with superior clinical outcomes, possess fused networks that support efficient oxidative phosphorylation (OXPHOS) and high spare respiratory capacity (SRC). Conversely, the chronic stress of the TME drives excessive *Drp1*-mediated fission, collapsing the network into a fragmented state linked to epigenetic locking and exhaustion (10).

Therefore, the next frontier in CAR-T engineering lies in supercharging this organelle. This mini-review synthesizes emerging strategies from 2024–2026, focusing on three cutting-edge domains: (1) targeting the metabolic-epigenetic axis (e.g., *P4HA1* inhibition) to unlock stemness, (2) genetic circuit engineering for hypoxic adaptation (Envirotune), and (3) the advent of organelle medicine via intercellular mitochondrial transfer (11, 12). By viewing the mitochondrion as a tunable biologic engine, we explore how restoring metabolic fitness can empower CAR-T cells to overcome solid tumor barriers. A comprehensive summary of these emerging strategies, including their mechanisms and functional impacts, is provided in **Table 1**.

**Table 1:** Emerging mitochondrial engineering strategies for next-generation CAR-T cell therapy.

Engineering domain	Target/platform	Mechanism of action	Metabolic and functional impact	Key references
Metabolic-Epigenetic Reprogramming	*P4HA1* Inhibition	Prevents the pathological translocation of *P4HA1* to mitochondria, blocking the conversion of α-KG to the oncometabolite succinate.	Restores α-KG levels to reactivate TET/JmjC demethylases; unlocks chromatin at stemness loci (*TCF7*) to reverse exhaustion.	(13)
Synthetic Biology (Environmental Sensing)	Envirotune(5V2-HRE + *SLC38A2*)	Utilizes a hypoxia-responsive promoter (5V2-HRE) upstream of a CMV minimal promoter to drive *SLC38A2* expression specifically in the TME.	Facilitates glutamine anaplerosis to fuel the TCA cycle under hypoxia; minimizes basal expression (“leakiness”) in normoxic tissues.	(14)
Organelle Medicine (Intercellular Transfer)	TNT-Mediated Transfer (Talin-2)	Facilitates the physical transfer of healthy mitochondria from stromal cells to T cells via Talin-2 stabilized tunneling nanotubes (TNTs).	Directly increases mitochondrial mass and Spare Respiratory Capacity (SRC); “supercharges” T cells for enhanced tumor infiltration.	(11)
Organelle Medicine (Device-Assisted)	MitoPunch/MitoCeption	Employs mechanical pressure (MitoPunch) or centrifugation (MitoCeption) to deliver isolated mitochondria, bypassing/utilizing endocytosis, respectively.	Enables scalable “mitochondrial doping” during manufacturing; restores respiration in dysfunction-prone cells.	(15, 16)
Genomic Optimization (CRISPR Screens)	*RHOG* Knockout	Inhibits trogocytosis (extraction of antigen from tumor cells), preventing CAR-T cells from acquiring and displaying tumor antigens.	Averts fratricide (sibling killing) and prevents chronic tonic signaling; preserves the effector pool density.	(17)
Genomic Optimization (CRISPR Screens)	*CDKN1B* (p27^Kip1^) Knockout	Removes the stress-induced cell cycle inhibitor p27^Kip1^, decoupling environmental stress from the G1/S checkpoint.	Prevents cell cycle arrest; maintains cells in a hyper-proliferative state despite chronic TME stress.	(18)
Structural Dynamics	*Opa1* Overexpression/*Drp1* Modulation	Promotes mitochondrial fusion (*Opa1*) or prevents excessive fission (*Drp1*) to maintain network integrity.	Enhances ETC supercomplex assembly and efficiency; imprints a central memory (TCM​) phenotype.	(19, 20)
Safety & Diagnostics	MitoR Algorithm	A computational tool using Poisson-Gamma mixture models to distinguish endogenous vs. exogenous mitochondrial transcriptional signatures.	Identifies patients at risk of therapeutic failure due to mitochondrial theft (“hijacking”); guides patient stratification.	(21)

## 2 The metabolic-epigenetic nexus: *P4HA1* and the succinate trap

While metabolic insufficiency has long been recognized as a hallmark of T cell exhaustion, the precise molecular mechanisms linking metabolic inputs to epigenetic stability have remained elusive (22, 23). A recently identified “metabolic-epigenetic checkpoint” driven by Prolyl 4-hydroxylase subunit alpha 1 (*P4HA1*) fundamentally alters our understanding of how the tumor microenvironment (TME) enforces permanent T cell dysfunction (13, 24).

### 2.1 *P4HA1*: an unlikely culprit in the mitochondria

*P4HA1* is canonically an endoplasmic reticulum (ER)-resident enzyme for collagen prolyl hydroxylation. However, under hypoxic and inflammatory conditions in solid tumors, *P4HA1* undergoes pathological translocation to the mitochondrial matrix of CD8+ T cells (13). There, it consumes α-ketoglutarate (α-KG), a key TCA cycle intermediate, to hydroxylate substrates, generating succinate as a byproduct (13). This depletes bioenergetic fuels while accumulating immunosuppressive oncometabolites (24, 25).

### 2.2 The succinate trap and epigenetic locking

This metabolic shift also impacts chromatin regulation. α-KG is an essential cofactor for TET DNA demethylases and JmjC histone demethylases (26, 27), and succinate competitively inhibits these enzymes (24). The resulting high succinate/low α-KG ratio creates a “succinate trap”, driving DNA hypermethylation and repressive histone marks (e.g., H3K9me3) at stemness loci such as *TCF7* (13, 28). This “epigenetic lock” fixes T cells in terminal exhaustion resistant to checkpoint blockade (23).

### 2.3 Therapeutic implications: restoring progenitor potential

Crucially, targeting the *P4HA1* axis offers a strategy to reverse this deep-seated dysfunction. Genetic ablation or pharmacological inhibition of *P4HA1* restores intracellular α-KG levels, reactivates TET/JmjC demethylases, and unlocks the chromatin at stemness-associated loci (13). This metabolic reprogramming promotes the expansion of TCF1^+^ progenitor CD8+ T cells, a subset critical for sustaining long-term antitumor immunity and responsiveness to PD-1 blockade (13, 29). These findings suggest that next-generation mitochondrial engineering must go beyond enhancing ATP production to explicitly correcting the accumulation of immunosuppressive metabolites like succinate (25). However, *P4HA1* translocation has been characterized primarily in specific murine models (13); whether it occurs universally across diverse solid tumors remains unclear. Clinical validation in patient-derived samples is lacking, and the effects of modulating the succinate/α-KG ratio on other TME-resident immune populations (e.g., Tregs, macrophages) warrant investigation.

## 3 Envirotune-CAR-T: turning hypoxic hostility into a metabolic advantage

While targeting intrinsic checkpoints like *P4HA1* restores epigenetic potential, CAR-T cells must also actively compete for nutrients in the resource-depleted tumor microenvironment (TME) (1, 30). Solid tumors present a tripartite liability of hypoxia, glucose deprivation, and acidosis. Conventional CAR-T cells, typically expanded under normoxic conditions *ex vivo*, often fail to adapt to this hostile landscape in patients. To overcome this, a novel “logic-gated” platform termed Envirotune-CAR-T was recently developed, which engineers T cells to sense hypoxic stress and respond with a synchronized metabolic reinforcement (14).

### 3.1 The “hypoxia-and-metabolism” logic gate

The Envirotune platform employs a synthetic promoter, 5V2-HRE, incorporating five VEGF-derived hypoxia response elements (HREs) upstream of a CMV minimal promoter (14). This architecture minimizes normoxic leakiness—a common flaw with strong constitutive promoters (31, 32)—restricting expression to the hypoxic tumor core (~1% O_2_). The 5V2-HRE drives co-expression of the CAR (targeting Mesothelin) and *SLC38A2* (a high-affinity glutamine transporter) via a P2A peptide, creating a biological “AND” gate activated only upon simultaneous antigen recognition and hypoxia detection (14, 33).

### 3.2 Rewiring metabolism via glutamine anaplerosis

The metabolic rationale for overexpressing *SLC38A2* is to counteract the “glutamine steal” imposed by tumor cells (34). In parallel, SLC38A2-mediated glutamine signaling has been established as an intercellular metabolic checkpoint in type-1 conventional dendritic cells (cDC1s), where it dictates antigen-specific CD8+ T cell priming and antitumor immunity (35). These complementary findings underscore the broader translational rationale for SLC38A2-based metabolic rewiring across multiple immune compartments. Under hypoxic conditions, T cells typically downregulate nutrient transporters as a survival mechanism, inadvertently starving themselves. Envirotune-CAR-T cells, however, are programmed to upregulate *SLC38A2* specifically under hypoxia (14). This forced influx of glutamine fuels the TCA cycle through anaplerosis (the conversion of glutamine to glutamate and then to α-ketoglutarate), bypassing the glycolytic block often seen in glucose-deprived tumors (36). Single-cell transcriptomic analyses have further revealed that the XBP1–*SLC38A2* axis serves as a critical metabolic regulator of cytotoxic T lymphocytes in tumor settings such as multiple myeloma, where XBP1-mediated suppression of *SLC38A2* directly compromises glutamine uptake and effector function (37).

Recent data demonstrates that this intervention fundamentally alters the bioenergetic profile of CAR-T cells. Even under stringent hypoxic conditions, Envirotune cells maintained high Basal Respiration and Spare Respiratory Capacity (SRC), metrics directly correlated with *in vivo* persistence (38, 39). Furthermore, the enhanced glutamine uptake supported the biosynthesis of glutathione (GSH), equipping the T cells with a superior antioxidant defense against the ROS-rich TME (40). By converting the hypoxic barrier into a trigger for metabolic armoring, this strategy exemplifies the next generation of context-aware cell therapies.

Important limitations remain, however. Complete elimination of normoxic leakiness has not been demonstrated, and even low-level off-target *SLC38A2* expression could cause systemic glutamine competition. Moreover, intratumoral hypoxia is spatially and temporally heterogeneous, potentially limiting Envirotune activation to only the deepest hypoxic niches. The long-term stability of synthetic promoters under chronic hypoxic stress and the translatability of the 1% O_2_ threshold to human tumors also require further evaluation.

## 4 Organelle medicine: intercellular mitochondrial transfer as a therapeutic modality

Organelle medicine—the physical transplantation of whole organelles—represents an emerging frontier in immunotherapy. Transferring healthy mitochondria into exhausted CAR-T cells can directly revitalize their metabolic function, circumventing endogenous repair limitations (41, 42).

### 4.1 Natural supercharging via tunneling nanotubes

BMSCs donate mitochondria to CD8+ T cells via tunneling nanotubes (TNTs) stabilized by Talin-2 (11, 43). Recipient “Mito+ T cells” exhibit increased basal respiration, superior SRC, and resistance to exhaustion under chronic antigen stress (44, 45). Beyond CD8+ T cells, mitochondrial transfer from MSCs has also been shown to modulate CD4+ T helper cell metabolism, including the suppression of IL-17 production and the promotion of Th17-to-Treg interconversion in autoimmune contexts (46), highlighting the broader immunometabolic consequences of intercellular organelle exchange.

### 4.2 Engineering synthetic delivery: methodological divergence

Translating this into scalable protocols presents challenges. MitoCeption uses centrifugation-based endocytic uptake of isolated mitochondria but risks lysosomal degradation of transferred organelles (16, 47). MitoPunch instead employs pressure-driven delivery directly into the cytoplasm, bypassing endocytosis and enabling simultaneous treatment of >10^5^ cells (15) and has further been shown to support stable transplantation of human mitochondrial DNA, generating non-immortal recipient clones with restored oxidative phosphorylation (48). These methods offer distinct trade-offs between accessibility and delivery efficiency for clinical manufacturing.

Critically, the immunogenicity of allogeneic mitochondrial DNA (mtDNA) poses a significant concern. As a DAMP, released mtDNA activates neutrophils via formyl peptide receptor-1 and TLR9 (49) and engages the cGAS-STING and NLRP3 inflammasome pathways to drive type I interferon and IL-1β responses (50), potentially exacerbating inflammation rather than enhancing antitumor function.

### 4.3 Current research gaps: the threat of mitochondrial hijacking

A critical controversy concerns the bidirectional nature of TNTs. While stromal-to-T cell transfer is well-established (11, 45), reverse mitochondrial hijacking by tumor cells remains an emerging hypothesis with preliminary supporting evidence. In the TME, aggressive tumor cells can hijack the TNT machinery to steal healthy mitochondria from infiltrating T cells, leaving the immune cells metabolically depleted—a process termed “mitochondrial hijacking” (43, 51, 52).

Direct evidence for tumor-to-T cell mitochondrial theft has been reported in breast cancer (43) and glioblastoma (53) models. However, generalizability across tumor types and clinical significance remain unclear. Notably, Miro1—the key regulator of intercellular mitochondrial transport—mediates strictly unidirectional transfer from mesenchymal to epithelial cells, with no detectable reverse transfer even under stress (54), suggesting hijacking depends on specific tumor-intrinsic factors rather than being a universal phenomenon.

To address this, MitoR (21) utilizes a Poisson-Gamma mixture model to analyze single-cell RNA sequencing (scRNA-seq) data. Unlike linear models such as MERCI (55, 56), which often fail to capture the complex noise of single-cell technologies (57), MitoR explicitly models the sparsity (high dropout rates) and over-dispersion inherent in scRNA-seq data. Crucially, rather than relying on indirect signaling markers (e.g., SASP), MitoR statistically discriminates between endogenous and exogenous mitochondrial transcriptional signatures. Assigning a probability score (θ) to mitochondrial reads, it enables the precise identification of tumor cells that have acquired T-cell mitochondria, thereby stratifying patients who may require TNT inhibitors to prevent therapeutic mitochondrial theft.

## 5 Genomic optimization: unveiling metabolic guardians via *in vivo* CRISPR screens

While targeting extrinsic metabolic checkpoints like *P4HA1* offers one avenue for rejuvenation, the intrinsic genetic wiring of CAR-T cells ultimately dictates their persistence (58). Historically, target discovery relied on candidate gene approaches limited by existing knowledge. However, unbiased, genome-wide *in vivo* CRISPR screening has recently shifted this paradigm by systematically mapping the genetic regulators of CAR-T persistence (59, 60). Removing specific “genetic brakes” identified through these screens can profoundly preserve functional competence by preventing the onset of terminal exhaustion and aberrant cell cycle arrest.

Genome-wide screening using the high-throughput CELLFIE platform identified gene knockouts that enhance CAR-T potency against leukemia (17). A key target is *RHOG*, encoding a Rac-related GTPase involved in actin remodeling. *RHOG* deletion decouples cytotoxicity from trogocytosis (61), serving a dual function: it prevents antigen stripping from tumor surfaces (averting immune escape) and blocks CAR-T cells from acquiring and displaying tumor antigens on their own surface, thereby preventing fratricide while preserving killing capacity (17).

Furthermore, removing *FAS* (CD95) shields CAR-T cells from activation-induced cell death (AICD) (62, 63). The dual knockout of *RHOG* and *FAS* creates a ‘super-soldier’ phenotype exhibiting superior expansion kinetics, demonstrating that combining structural modulation (trogocytosis blockade) with apoptotic resistance confers a robust survival advantage (17).

Temporal analysis using the ‘Mario’ sgRNA library in a multiple myeloma model revealed that genetic requirements for survival evolve dynamically (18). While *PTPN2* and *SOCS1* control early expansion (64–66), *CDKN1B* (encoding p27^Kip1^) restricts long-term persistence (Day 21+) (18). Biologically, p27^Kip1^ functions as a critical checkpoint inhibitor that arrests the cell cycle at the G1/S transition. In the tumor microenvironment, chronic stress triggers a DNA damage response that upregulates p27^Kip1^, forcing cells into a non-proliferative state. Consequently, *CDKN1B* ablation enables CAR-T cells to decouple environmental stress from cell cycle arrest, allowing them to maintain proliferative capacity late into the tumor challenge—effectively transforming transient effectors into long-term guardians (18). Similar proliferative fitness determinants have been observed in chronic lymphocytic leukemia (CLL) (67).

Despite these advances, a major research gap remains regarding the safety of these “unbraked” T cells. Removing tumor suppressors like *FAS* or *CDKN1B* raises theoretical concerns about autonomous proliferation or lymphomagenesis (68). Although it is demonstrated that *CDKN1B*-knockout cells remain cytokine-dependent for survival, rigorous long-term safety studies are required before clinical translation. Future developments will likely focus on logic-gated CRISPR editing, where these deletions are induced only within the tumor site to minimize systemic risk (69).

Furthermore, *RHOG* and *CDKN1B* were identified in leukemia and myeloma models, respectively; their relevance to solid tumors remains to be validated. Conflicting evidence also exists regarding *FAS*: while deletion protects against AICD, FAS-FasL signaling contributes to direct tumor killing in certain contexts (62), suggesting ablation may reduce cytotoxicity against FasL-sensitive tumors.

## 6 Mitochondrial dynamics: structuring the metabolic engine for longevity

Beyond enzymatic activity, the gross morphology of the mitochondrial network—governed by the opposing forces of fusion and fission—serves as a physical rheostat for T cell differentiation (19, 70). Recent evidence indicates that manipulating these structural dynamics offers a potent avenue to enforce a memory phenotype and prevent the metabolic burnout often observed in CAR-T cells.

Mitochondrial fusion, mediated by *Opa1*, is a hallmark of stem-like central memory T cells (TCM). Fused networks promote ETC supercomplex assembly, efficient OXPHOS, and cytochrome c sequestration (71). Enforcing fusion via *Opa1* overexpression or pharmacological agents imprints a memory-like metabolic profile with high SRC, supporting long-term persistence (19, 72).

Conversely, mitochondrial fission, driven by Dynamin-related protein 1 (*Drp1*), presents a significant scientific controversy often described as the “*Drp1* Paradox”. On one hand, transient *Drp1*-mediated fission is physiologically indispensable during the initial phase of T cell activation to support rapid clonal expansion and the equal segregation of mitochondria into daughter cells (73, 74). Complete ablation of *Drp1* can therefore cripple the initial immune response and impair tumor infiltration. On the other hand, under the chronic antigen stimulation characteristic of solid tumors, *Drp1* becomes pathologically hyperactivated (75, 76). This drives continuous, excessive fission, resulting in a fragmented mitochondrial network that is incapable of efficient respiration and prone to ROS generation—a structural state linked to epigenetic locking and terminal exhaustion (20, 77, 78).

Resolving this paradox requires a nuanced approach rather than a binary blockade. The current consensus suggests that next-generation engineering must aim to tune mitochondrial dynamics. For instance, rather than permanent genetic ablation, pharmacological agents like Mdivi-1 (a *Drp1* inhibitor) are being explored to dampen excessive fission specifically during the *ex vivo* expansion phase (79). This metabolic priming steers CAR-T cells toward a fused, memory phenotype prior to infusion, allowing them to retain their proliferative potential while resisting fragmentation upon encountering the tumor (80).

Notably, Mdivi-1 functions primarily as a reversible complex I inhibitor rather than a direct *Drp1* antagonist (79), complicating mechanistic interpretation. The optimal fusion-fission balance is likely patient- and tumor-specific, necessitating predictive biomarkers for clinical application.

**Figure 1:**
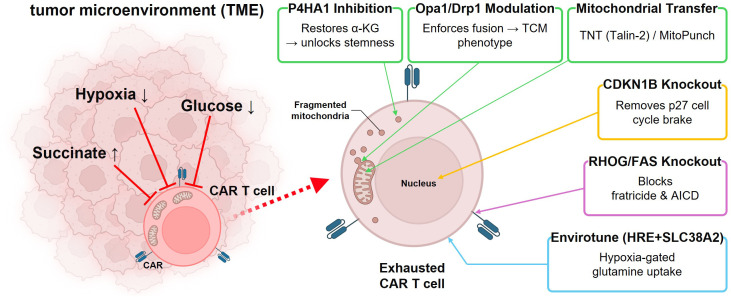
Overview of mitochondrial engineering strategies for overcoming TME-driven CAR-T cell exhaustion. (Left) CAR-T cells infiltrating the solid tumor encounter a metabolically hostile TME characterized by hypoxia, glucose deprivation, and succinate accumulation. (Right) Zoom-in of an exhausted CAR-T cell showing fragmented mitochondria and the six engineering strategies targeting specific cellular compartments: *P4HA1* inhibition and *Opa1*/*Drp1* modulation target mitochondria; mitochondrial transfer delivers healthy organelles from external sources; Envirotune enhances glutamine uptake at the cell membrane; *CDKN1B* knockout relieves cell cycle arrest in the nucleus; *RHOG*/*FAS* knockout prevents fratricide and AICD at the cell surface.

A schematic overview of the TME metabolic battlefield and the points of action of each mitochondrial engineering strategy is provided in **Figure 1**.

## 7 Discussion: toward multiplex metabolic engineering in the clinic

These strategies underscore a paradigm shift: CAR-T exhaustion is a downstream consequence of mitochondrial collapse, not merely a transcriptional state. The TME’s complexity likely necessitates combinatorial approaches coupling intrinsic genetic rewiring with extrinsic organelle augmentation.

For instance, coupling Envirotune (*SLC38A2*) (14) with *RHOG* ablation (17) could provide metabolic fuel while insulating against exhaustion. Similarly, combining *P4HA1* inhibitors with *Opa1* overexpression (13, 19) could ensure both epigenetic unlocking and fused mitochondrial networks.

These engineering approaches raise important safety concerns. Forced *SLC38A2* overexpression risks systemic glutamine competition, while enhanced mitochondrial respiration may generate excessive ROS. CRISPR-mediated deletion of tumor suppressors (*FAS, CDKN1B*) theoretically increases lymphoproliferation risk, though edited cells remain cytokine-dependent (18); off-target editing and structural variants remain concerns (68). For organelle therapies, allogeneic mtDNA can activate innate immunity through FPR1/TLR9 (49) and cGAS-STING/NLRP3 (50) pathways. Integration of inducible safety switches (e.g., iCasp9) into metabolically engineered products is therefore essential.

Translational feasibility varies by modality. Pharmacological approaches (*P4HA1* inhibitors, Mdivi-1) are the most Good Manufacturing Practice (GMP)-scalable, requiring no genetic modification. CRISPR-based strategies must meet stringent regulatory requirements, including comprehensive off-target analysis, validated clearance of residual Cas9/gRNA, and a recommended 15-year post-treatment follow-up (81). Organelle medicine faces the greatest hurdles: TNT-mediated transfer is inherently variable, and synthetic delivery (MitoPunch) demands specialized equipment and rigorous mitochondrial quality control. A comparative assessment of these strategies is provided in **Table 2**.

**Table 2:** Qualitative comparative assessment of mitochondrial engineering strategies.

Strategy	Persistence	Tumor control	Memory phenotype	Scalability	Clinical readiness	Key reference
*P4HA1* Inhibition	++	+	+++	High	Preclinical	(13)
Envirotune (HRE+*SLC38A2*)	++	++	+	Medium	Preclinical	(14)
TNT Transfer (Talin-2)	+++	++	++	Low	Early preclinical	(11, 43)
MitoPunch/MitoCeption	++	+	++	Medium	Early preclinical	(15, 16)
*RHOG/FAS* Knockout	++	+++	+	High	Preclinical	(17)
*CDKN1B* Knockout	+++	++	++	High	Preclinical	(18)
*Opa1*/Mdivi-1	++	+	+++	High	Preclinical	(19, 20)

+, modest; ++, moderate; +++, strong. Based on available preclinical data.

A further concern is mitochondrial hijacking—tumor cells exploiting TNTs to steal supercharged organelles (43, 53, 82). Predictive tools such as MitoR (21) could identify at-risk patients, while strategies for metabolic containment (e.g., cytoskeletal anchoring via reinforced Talin-2 linkages) warrant development.

The next generation of CAR-T cells will be defined not just by what antigen they see, but by how they fuel the fight. By systematically repairing the metabolic-epigenetic axis, engineering hypoxia resistance, and physically replenishing mitochondrial mass, we can transform CAR-T cells from transient effectors into durable, metabolically fit guardians capable of overcoming the hostile frontiers of solid tumors.
